# Precise Decision-Making and Adaptive Response Strategies Based on the Situations of Stress During the Coronavirus Disease 2019 (COVID-19) Pandemic

**DOI:** 10.3389/fpubh.2020.00364

**Published:** 2020-07-07

**Authors:** Weifeng Shen

**Affiliations:** ^1^Department of Emergency Medicine, The Second Affiliated Hospital, Zhejiang University School of Medicine, Hangzhou, China; ^2^Institute of Emergency Medicine, Zhejiang University, Hangzhou, China

**Keywords:** COVID-19, complex emergencies, coping strategies, decision making, public health

## Introduction

Currently, coronavirus disease 2019 (COVID-19) has developed into a worldwide pandemic, and the number of COVID-19 cases is growing rapidly. By May 15, 2020, there were more than 4 million confirmed cases of COVID-19 and more than 297,000 deaths reported globally (1). Measures, such as social distancing, travel restrictions, school closures, and augment of medical capacity have been taken to try to control the COVID-19 pandemic, but the global pandemic is still spreading in general. The risk of an epidemic rebound and even further spread remains even after the epidemic has come under control in some countries and regions. Additionally, the pandemic has turned increasingly more complex. Suffering from the strong impact of the COVID-19 pandemic, the current healthcare system as the core system of the prevention and treatment of the COVID-19 pandemic has been severely affected (2). If the health care system becomes overloaded or collapses, it will seriously affect the medical care of patients with COVID-19, which will then cause even greater risks for society as a whole (3). Therefore, as a new perspective, the explosive situation of stress the healthcare system is currently experiencing should be considered during the decision-making and development of a response to the COVID-19 pandemic.

## Discussion

Decision-making and response are based on the assessment of the COVID-19 pandemic, which can be empirically and actually measured. However, at the beginning of the epidemic, the number of cases of COVID-19 were low, and the “clues” were easy to miss. In addition, it is possible that the extent of the danger was not fully exposed in some areas where the epidemic is mild. We should be alert to the “reverse rescue” trap. It is necessary to have a forward-looking assessment of the risks, dynamic predictions, medical needs, and effects of COVID-19 prevention and control. Mathematical models and computer simulations can play an active role (4–6), but the estimated values generated by mathematical models and computer simulations are quite different from the actual situation due to the unpredictable and volatile nature of the pandemic.

Decision-making and response should not only address the existing situation of the COVID-19 pandemic but also be prepared in advance to deal with the challenge that pertains to the potential medical surge caused by COVID-19 for the period of time following the outbreak and spread of the pandemic (7, 8). Comprehensive decision-making for the COVID-19 pandemic should not stop at the survey of current risks and demands, but it should predict the risks and demands for the following period, not only to assess the demands placed on the health care system by the COVID-19 pandemic but also to evaluate the supply side of prevention, control, and response. The stress situation of the healthcare system is the specific consequence of the balance between the demand side and the supply side, and decision-making guided by identifying the stress situation of healthcare system will be more precise and comprehensive. The stress situation of the healthcare system can be presented from the three dimensions of medical surge, medical capacity, and system vulnerability. The spread of infection of COVID-19 in healthcare workers is an essential factor of vulnerability (9). A total of 1,716 healthcare workers were among the confirmed cases in the early phase of the COVID-19 pandemic in China (10). After that, due to increased awareness of this problem, the workforce of healthcare workers received strict protection. Of the more than 42,000 healthcare workers who traveled to Hubei province, China, to support for responding to COVID-19 pandemic there, none were infected (11).

In short, based on the assessment of medical surge, medical capacity, and system vulnerability, we can fully evaluate the stress situation of the health care system during the COVID-19 pandemic.

**Figure 1:**
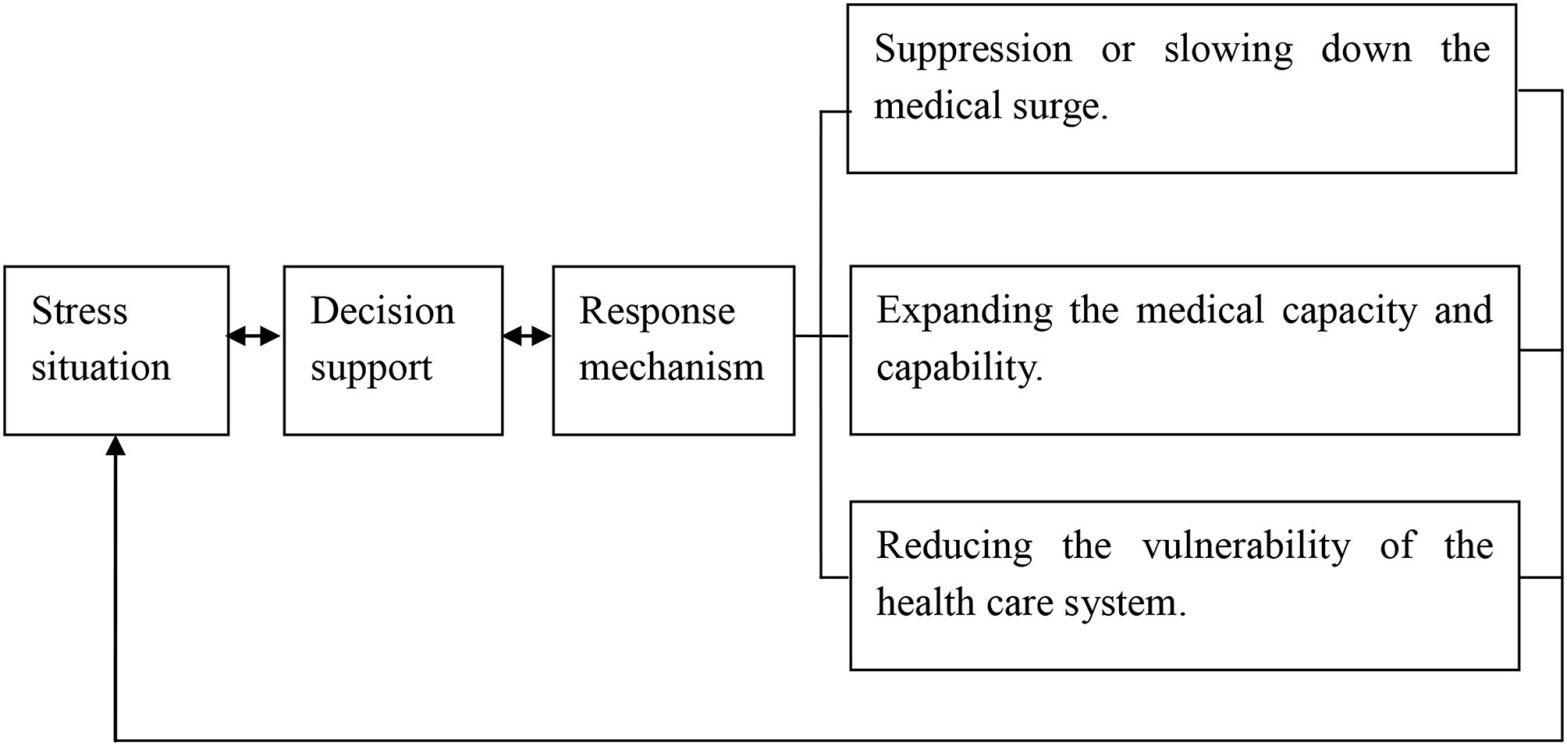
Coupling model of “stress situation, decision support, and response mechanism”.

According to the assessment of stress situation of the healthcare system during the COVID-19 pandemic, it can be considered as a promising method by using the novel coupling model for decision-making in a macro perspective, as shown in Figure 1.

Decision-making of COVID-19 prevention and control in a macro perspective can be considered from three different aspects. First, in the direction of suppression or slowing down the medical surge, the main practical measures include social distancing, wearing masks, lock-down policy, close-off management, control in advance, and isolation for the confirmed and suspected cases before the successful development of COVID-19 vaccine (12–17). Second, in the direction of expanding the medical capacity and capability, we should launch or upgrade the level of response, external support, centralized response, and reasonably adjust care standards or simplify care procedures. In addition to conventional measures, unconventional measures should be actively taken to relieve the stress on the health care system. In the city of Wuhan, the area worst affected by the COVID-19 pandemic in China, two large temporary hospitals were built for the centralized treatment of severe cases of COVID-19, and dozens of exhibition halls and stadiums were converted into “Fangcang” shelter hospitals for treatment of mild cases of COVID-19, which gradually relieved the stress of the shortage of beds in hospitals (18). The rate of development of the COVID-19 pandemic frequently exceeds predictions or expectations. Once the COVID-19 cases begin to increase rapidly, particularly in community transmission occurrences, external support needs to be initiated as soon as possible. In addition to beds, ventilators, and personal protective equipment, the lack of health care workers is a key variable that intensifies stress. For this reason, more than 42,000 health care workers throughout China went to support the workforce in the Hubei province, the area most seriously affected, and the policy of nationwide response, “one province supports one region,” was implemented (19). It is aimed at taking over the individual hospital or ward within the integrated system and providing integrated support at the peak of the COVID-19 pandemic. The “four centralized responses” of “centralizing patients, experts, resources, and care” is an unconventional strategy for allocation of scarce medical resource on the basis of demand, which has achieved remarkable effects in the response to the COVID-19 pandemic in China (20). Following the principle of “maximizing the number of cases with the maximizing benefits,” it can be carefully and reasonably adjusted for the care standards and procedures for COVID-19 cases, particularly in critical supply shortages (21). Third, in the direction of reducing the vulnerability of the health care system, joint prevention and control mechanism, public participation prevention and control mechanism, coordination between epidemic prevention system and treatment system, coordination between response system and logistics system, multi-disciplinary combination, and international cooperation can play an active role in response to the COVID-19 pandemic (22).

## Limitations

With any new proposals, especially those that immediately involve the entire population, there will be concerns raised as to its limitations. Firstly, the current proposals of decision-making framework for responding to the COVID-19 pandemic in this paper are still preliminary. The quantitative identification and description of the stress situation of health care system during the COVID-19 pandemic needs to be further studied. Secondly, this article is based on the current experience and lessons learned from the COVID-19 prevention and treatment. With the continuous accumulation of experience in COVID-19 prevention and treatment, we need to collect more evidence and data to improve the proposed views.

## Conclusion

In the specific scenario of the COVID-19 pandemic, social distancing, wearing masks, lock-down policy, close-off management, advance control, and isolation of the confirmed and suspected cases are considered effective prevention and control measures. Launching or upgrading the level of response, external support, centralized response, and reasonably adjusting care standards or simplifying care procedures are also important aspects of epidemic response. Additionally, reducing the vulnerability of the healthcare system should not be ignored. From the previous perform of COVID-19 pandemic control and treatment, we can see that efforts for a single dimension cannot effectively alleviate the stress of the health care system during the COVID-19 pandemic, effective efforts should be made for the three dimensions including the medical surge, the medical capacity and the vulnerability of the health care system. In summary, decision-making of COVID-19 prevention and control in a macro perspective can be considered from three different aspects: suppression or slowing down the medical surge, expanding the medical capacity and capability, and reducing the vulnerability of the healthcare system. These aspects can be incorporated into future policies and decision-making during the COVID-19 pandemic and the current decision-making framework need to be improved by summarizing more lessons learned from the COVID-19 pandemic.

## Author Contributions

WS contributed to the conception of the study and wrote the manuscript.

## Conflict of Interest

The author declares that the research was conducted in the absence of any commercial or financial relationships that could be construed as a potential conflict of interest.
